# Population Dynamics of a Declining White-Tailed Deer Population in the Southern Appalachian Region of the United States

**DOI:** 10.3390/ani13233675

**Published:** 2023-11-28

**Authors:** Adam C. Edge, Jacalyn P. Rosenberger, Charlie H. Killmaster, Kristina L. Johannsen, David A. Osborn, Karl V. Miller, Gino J. D’Angelo

**Affiliations:** 1 Daniel B. Warnell School of Forestry and Natural Resources, University of Georgia, 180 E Green Street, Athens, GA 30602, USA; jackie.rosenberger@dwr.virginia.gov (J.P.R.); osborn@warnell.uga.edu (D.A.O.); kmiller@warnell.uga.edu (K.V.M.); gdangelo@uga.edu (G.J.D.); 2 Game Management Section, Wildlife Resources Division, Georgia Department of Natural Resources, 2067 US-278, Social Circle, GA 30025, USA; charlie.killmaster@dnr.ga.gov (C.H.K.); tina.johannsen@dnr.ga.gov (K.L.J.)

**Keywords:** game management, harvest regulations, *Odocoileus virginianus*, population dynamics, Southern Appalachians, stage-structured population model, white-tailed deer, vital rates

## Abstract

**Simple Summary:**

We examined a white-tailed deer (*Odocoileus virginianus*) population with evidence of decline in the Southern Appalachian region of the United States. In the Chattahoochee National Forest (CNF) in northern Georgia, deer harvest numbers and hunter success rates have drastically reduced over the last few decades, suggesting a decline in deer density. Low fawn survival (16%) was also recently recorded, prompting further concern regarding deer population sustainability. In the CNF, we radio-collared 14 yearling and 45 adult female white-tailed deer along with 71 fawns during 2018–2020 to estimate the annual survival and fecundity of each age class. We modeled future population growth (λ) over 10 years to evaluate the current rate of decline and various other management scenarios. Projections indicated that the white-tailed deer population will decline by an average of 4.0% annually under current conditions, and only scenarios that incorporated both antlerless harvest restrictions and improved fawn survival will lead to positive population growth. Thus, these approaches should be emphasized in future management plans within the Southern Appalachian region to facilitate recovery. This methodology can be applied by other wildlife managers with knowledge of site-specific vital rates to inform potential management strategies.

**Abstract:**

Although generally abundant, white-tailed deer (*Odocoileus virginianus*) populations in the southeastern United States have recently experienced several localized declines attributed to reduced fawn recruitment following the establishment of coyotes (*Canis latrans*). The Southern Appalachians is a mountainous region suggested to be experiencing white-tailed deer declines, as harvest numbers and hunter success rates have substantially decreased in northern Georgia since 1979. Low fawn survival (16%) was also recently documented in the Chattahoochee National Forest (CNF) in northern Georgia, necessitating further examination. We radio-collared 14 yearling and 45 adult female white-tailed deer along with 71 fawns during 2018–2020 in the CNF to estimate field-based vital rates (i.e., survival and fecundity) and parameterize stage-structured population models. We projected population growth rates (λ) over 10 years to evaluate the current rate of decline and various other management scenarios. Our results indicated that the observed population would decline by an average of 4.0% annually (λ = 0.960) under current conditions. Only scenarios including antlerless harvest restrictions in addition to improved fawn survival resulted in positive growth (λ = 1.019, 1.085), suggesting these measures are likely necessary for population recovery in the region. This approach can be applied by wildlife managers to inform site-specific management strategies.

## 1. Introduction

In the southeastern United States (U.S.), white-tailed deer (*Odocoileus virginianus*) populations are generally considered to be abundant [1]. However, recent studies have suggested several white-tailed deer populations in the region are experiencing localized declines [2,3,4]. Documented reductions in fawn recruitment have been attributed to the range expansion of coyotes from the western U.S. to the southeastern states during the last few decades, as low fawn survival rates of 14% [3], 16% [4], 23% [5], 26% [6], and 29% [7] have been observed due primarily to coyote predation. Multiple predator removal studies have suggested coyotes may contribute to additive fawn mortality at some sites [8,9,10,11,12], with a lack of compensatory response by other mortality sources recorded by Kilgo et al. [10] during years of decreased coyote predation. Fawn mortality due to coyotes, in addition to other sources of site-specific mortality and environmental factors, may have long-term effects on the sustainability of white-tailed deer populations [2].

The Southern Appalachians is a forested mountain region within the southeastern U.S. where evidence has indicated concern regarding white-tailed deer population sustainability [4,13] and associated hunter retention [14]. In northern Georgia, the number of deer harvested and hunter success rates (i.e., males harvested/hunter/day) has decreased substantially in the Chattahoochee National Forest (CNF) since 1979 [13,14], indicating likely declines in deer density. The reduction in female harvest was partially due to increasingly restrictive antlerless harvest regulations as a strategy to promote deer population growth, but populations have seemingly failed to respond. A concurrent study within the CNF estimated a low fawn survival rate of 16% during 2018–2020 due primarily to predation, causing 82% of total mortalities, with natural causes being responsible for the remaining 18% [4]. In comparison, average fawn survival in contiguously forested landscapes across North America was reported at 41% [15]. Edge et al. [4] concluded that a changing predator context in an increasingly homogenous landscape was adversely impacting fawn recruitment in northern Georgia and leading to potential population decline. As opposed to most of the 20th century, the Southern Appalachian region currently accommodates a triumvirate of expanding predator populations that comprises coyotes, black bears (*Ursus americanus*), and bobcats (*Lynx rufus*), all of which are sources of fawn mortality [5,16,17]. Coyotes recently established populations after arriving in the mountainous region in the 1980s [18], while native black bears and bobcats have increased in abundance since that time [13,19]. Edge et al. [4] found that coyotes, black bears, and bobcats were responsible for a known 40%, 22%, and 13% of fawn mortalities in the CNF, respectively, with an additional 7% of mortalities due to an undetermined predator.

While predation is most often the immediate cause of death for neonatal white-tailed deer fawns, underlying factors can make fawns more or less susceptible to predation as reflected in the wide range of fawn survival rates found across their range (0.14–0.90; [15]). Fawn predation rates increase in landscapes of decreased habitat diversity and edge, which may lack refugia for fawns (e.g., large forest patches with limited edge habitats; [15,20,21]). Southern Appalachian forests have become progressively homogenous, with a lack of vegetative diversity [13,22]. In the CNF, 88% of forest stands are ≥61 years old, with little early successional habitat [13], due in part to suppression of natural fire regimes [22,23] and declining timber harvests in recent decades [24]. As forests mature, closed canopies block sunlight from reaching the forest floor, causing reduction of the understory vegetation [25] utilized by fawns to hide from predators.

A lack of quality forage in the understory can also fail to meet nutritional demands for reproductive females during gestation and lactation periods [26]. Poor habitat conditions can restrict female fecundity [27] and reduce maternal care [26], influencing population dynamics as a result. White-tailed deer productivity in the Southern Appalachians is typically lower relative to surrounding regions but has remained stable over time, with an average fetal rate of 1.47 fetuses/adult female recorded throughout the region in the 1980s [28] and an average birth rate of 1.43 fawns/female observed in northern Georgia during 2018–2020 [4]. Considering this consistency of reproductive rates over time and improving herd condition based on increasing mass, antler beam length, and antler beam diameter of yearling bucks [13], reduced fecundity is not likely a factor in population decline. However, reproductive productivity is seemingly capped due to the high acorn dependence of Southern Appalachian deer populations [28]. Yearling female fetal rates and deer condition of all sex and age classes were found to fluctuate in relation to the annual acorn (*Quercus* spp.) crop in the 1980s [28,29]. Further evidence from the Central Appalachians has suggested acorn availability can influence fawn survival as well [30]. Therefore, deer vital rates likely follow an episodic pattern due to the density-independent effects of annual acorn production [31]. Dependence on acorn availability may make deer populations in northern Georgia particularly susceptible to the combined effects of abundant predators and marginal habitat conditions [4].

Assessing the changes in age-specific vital rates of white-tailed deer populations driven by mechanisms of predation and habitat conditions is fundamental [32]. Therefore, understanding the population dynamics of this declining deer population by identifying which vital rates can efficiently change population growth is necessary to assess the potential recovery and outcomes of management actions such as antlerless harvest restrictions [33,34]. Robinson et al. [35] simulated age-based population models parameterized with vital rates collected from studies of white-tailed deer across the eastern U.S. to assess different levels of antlerless harvest on future population sustainability. They concluded that the reduction of antlerless harvest rates should offset the impacts of coyote predation on fawns under most circumstances. However, juvenile survival of ungulates can vary spatially and temporally due to environmental factors and predation effects [32,36,37]. Therefore, using local field-based vital rates is requisite for management purposes. For example, Chitwood et al. [38] developed life-stage matrix models using field-based vital rates to examine potential management actions for a declining white-tailed deer population in Fort Bragg, North Carolina. They concluded that restricting antlerless harvest alone would not induce deer population growth. Under different conditions, Peters et al. [39] used a similar modeling method with vital rates documented in the field and found that the white-tailed deer population of the Tensas River National Wildlife Refuge in Louisiana could sustain additional female harvest despite current fawn predation rates.

We evaluated the population dynamics of a declining white-tailed deer population within the Southern Appalachian region of northern Georgia on a landscape characterized by homogenous habitat structure and high predator abundances. Our objectives were to use field-based vital rate estimates to assess the rate of deer decline and simulate population trajectories under differing “what if” management scenarios. We compared population growth rates under five scenarios that included observed conditions and differing antlerless harvest and fawn survival rates. In doing so, we provided further examination of the utility of stage-structured population modeling for managers seeking to inform their own site-specific wildlife management strategies. We hypothesized that antlerless harvest restrictions alone would be insufficient to facilitate population recovery due to significantly low fawn survival. If antlerless harvest restrictions were found to be inadequate as an independent strategy, additional management actions would be required.

## 2. Materials and Methods

### 2.1. Study Site

The study landscape comprised 135 km^2^ of CNF lands (89%) and intermixed private property (11%) located within Fannin and Union counties in northern Georgia, USA. This area was located within the southern extent of the Southern Appalachian Mountains in the Blue Ridge physiographic province. Portions of the Blue Ridge (85 km^2^) and Coopers Creek (121 km^2^) Wildlife Management Areas (WMAs) made up the majority of the CNF included in our study area. While the WMAs were located on federal lands, game management was administered by the Georgia Department of Natural Resources (GDNR) in cooperation with the U.S. Forest Service (USFS). The study area was representative of public and private land ownership patterns typical of the Southern Appalachian region.

The study area was characterized by rugged topography and elevations ranging from 198–1458 m, including steep slopes and drainages. North-facing slopes were dominated by upland hardwoods (e.g., *Quercus alba*, *Q. montana*, *Q. rubra*, *Liriodendron tulipifera*, *Acer rubrum*, and *Carya* spp.), and south-facing slopes had a mix of hardwoods and pines (*Pinus* spp.; [40]). Mountain laurel (*Kalmia latifolia*) and rosebay rhododendron (*Rhododendron maximum*) formed dense patches in the understory and midstory [41]. Forest conditions were predominantly in later seral stages, with diminished understory vegetation. Private land holdings provided more open land cover in the form of old fields and pastures.

White-tailed deer were extirpated from northern Georgia in 1895, primarily from overhunting [42]. Restoration began in 1928, with the release of four deer into the Rock Creek Refuge (now Blue Ridge WMA; [43]), and continued thereafter, leading to the first managed deer hunt in 1940 [44]. Deer densities grew to an estimated density of 7 deer/km^2^ in 1953 [44], continued to grow to 6.2–8.5 deer/km^2^ in 1982 [45] after further restocking efforts [43], and reached 7.3–9.3 deer/km^2^ in 1993 [46] on the Blue Ridge and Coopers Creek WMAs. In the early 2000s, a steep downward trend in deer harvest was observed, and densities were recently estimated to range from 1.9–3.9 deer/km^2^ across the northern Georgia area [13]. As a result of observed low fawn survival in northern Georgia by Edge et al. [4], the USFS and GDNR eliminated all antlerless deer harvest on federal and state public lands in the northeastern part of the state in 2019 and 2020, respectively. Antlerless deer harvest was still permitted on private properties throughout the state during the entire study, but opportunity was limited. The statewide bag limit per hunter equaled 10 antlerless and 2 antlered deer per year.

### 2.2. Deer Capture and Monitoring

We captured female white-tailed deer ≥1.5 years of age from January to April 2018–2020 using rocket nets, drop nets, Clover traps [47], and dart projectors (Pneu-dart, Williamsport, PA, USA). We immobilized all deer with a 2-cc intramuscular injection of butorphanol, azaperone, and medetomidine (BAM; Wildlife Pharmaceuticals, Windsor, CO, USA; [48]). We GPS-collared all deer (Vectronic Aerospace Gmbh, Berlin, Germany) and inserted vaginal implant transmitters (VITs) into receptive females to facilitate fawn capture. No yearling females were able to receive VITs [4]. We categorized individuals as either yearlings or adults by tooth wear and replacement [49]. We reversed immobilized deer using 4.0 cc of atipamezole plus 0.5 cc of naltrexone (Wildlife Pharmaceuticals, Windsor, CO, USA; [48]) and released deer at the location of capture. We received mortality notifications via email from satellite communication once GPS-collar fixes were stationary for ≥8 h and monitored survival until the end of the 3-year study (Table S1). Subsequent fawn capture and monitoring methods were as described by Edge et al. [4].

### 2.3. Model Description and Parameters

**Figure 1:**
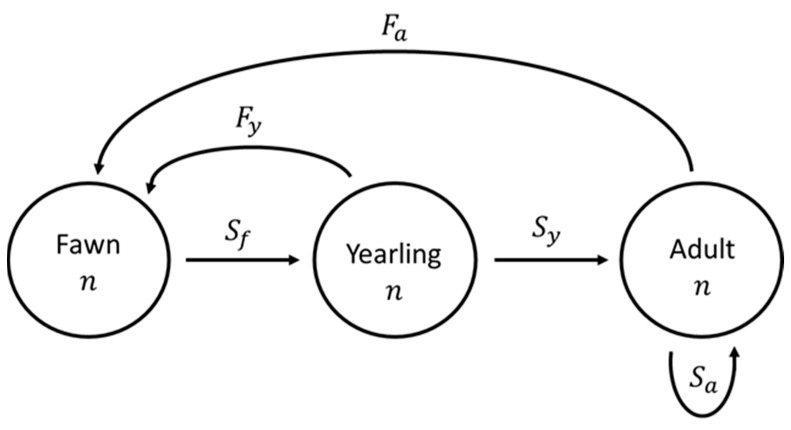
Abundance (*n*) progression of one age class to the next in a stage-structured population model used for female white-tailed deer (*Odocoileus virginianus*) with three stages—fawn (*f*), yearling (*y*), and adult (*a*)—in northern Georgia, USA, 2018–2020. Survival between stages is represented by *S*, and fecundity values are represented by *F*.

We used a stage-structured population model [50] including only female deer to project population growth rates 10 years into the future for multiple scenarios. Our model design was female-exclusive because the number of females is the best indicator of recruitment and concurrent long-term population growth [51]. The population model included three life stages: fawn (0–1 year old), yearling (1–2 years old), and adult (≥2 years old; Figure 1). Surviving fawns and yearlings transitioned to the next life stage after each time step, while surviving adults remained in the same stage for the duration of the 10-year projection. The matrix model was as follows:

$$A\times n_{t}=n_{t+1}$$

where $n_{t}$ is a vector of initial abundances n for each life stage at initial time t, and A is a 3 × 3 Leslie matrix of vital rates for each life stage that projects abundances in a vector for the following time period t+1, measured as one year. Matrix A followed the ensuing structure, including survival *S* and fecundity *F*:

$$A=\left[\begin{array}{ccc} 0 & F_{yearling} & F_{adult}\\ S_{fawn} & 0 & 0\\ 0 & S_{yearling} & S_{adult} \end{array}\right]$$

Our model followed assumptions similar to Chitwood et al. [38] and Peters et al. [39] to allow for comparison among studies. We assumed population growth of our deer population to be density-independent, as the declining white-tailed deer population in our study region showed no evidence of density-limiting effects [13]. We presumed geographic closure, as female deer are known to show high site fidelity [52,53]. Lastly, the fecundity rate parameters were assumed constant for each life stage [39]. There was no evidence for fawn reproduction on our study site [4,28]. Observed adult reproductive rates, comparing fetal and birth rates, have been observed to remain consistent year to year [28] and over recent decades [4], with no evidence of senescence in white-tailed deer [54]. Yearling reproductive rates were previously concluded to fluctuate annually based on acorn abundance [28], but limited observation of yearling reproduction in our study reduced our ability to include variation.

To populate the model, we used estimated vital rates from collared individuals in our field study. We estimated yearling and adult annual survival without covariates using Kaplan–Meier survival curves [55] generalized for the case of staggered entry [56] using the ‘survival’ package [57] in R software (version 4.1.3). To estimate the non-hunting adult survival rate, we censored hunter-related mortalities (*n* = 2) from the number of captured adults (*n* = 45) to account for the natural survival rate, assuming harvest was an additive source of mortality [39]. All yearlings in our study survived (*n* = 14), which was most likely an effect of the small sample size. The literature lacks additional estimates of mortality for yearling female deer in the southeastern U.S. from hunting and non-hunting sources, although typically it is assumed to be low [58]. Therefore, we substituted a yearling non-hunting survival estimate of 0.952 (95% CI = 0.866–1.000; *n* = 21), as recorded by Peters et al. [39] on a WMA in Louisiana, due to the similar predator composition to our study area and the utility of the provided confidence intervals. During concurrent research on our study site, fawn survival rates of 0.157 (95% CI = 0.091–0.273; *n* = 71) and 0.196 (95% CI = 0.096–0.403; *n* = 30) were documented from birth to 12 weeks of age using cumulative fawn data and a subset of VIT-captured fawn data, respectively [4]. We chose to use the lower fawn survival rate (0.157) for our population models to remain conservative, as overestimation of survival with the use of left-truncated data was not an issue, and the sample size used to calculate this rate was much higher [4]. Previous studies in the southeastern U.S. also used a 12-week fawn survival rate to represent the entire fawn stage, as mortality rates of older fawns (0.5–1.0 years of age) were insignificant [38,39]. Hunter harvest is expected to be the main mortality risk during the older fawn age period in the southeastern region, but no harvests of our tagged fawns were reported. We calculated fecundity as $F_{i}=(B_{i}\times S_{i})/2$, with $B_{i}$ representing the birth rate and $S_{i}$ signifying the annual survival rate of a specific life stage i [50]. To retain a female-only model structure, we calculated fecundity values as the percentage of individuals in a life stage that survived to give birth to female fawns [38], assuming a 1:1 male-to-female birth ratio [59]. The observed birth rate for adult females on our study area was 1.43 fawns/adult female (30 fawns captured via VIT from 21 collared adults) over the 3-year study period [4]. We acknowledge the possibility that we failed to capture sibling fawns in some instances, so the birth rate for adults is a conservative estimate. However, the birth rate we observed was consistent with the average fetal rate of 1.47 fetuses/adult female recorded by Wentworth et al. [28], providing us confidence in our estimate and the comparative use of the historic reproductive data. We were unable to accurately monitor births of yearling females, as they were not receptive of VITs during capture [4]. Thus, we used the only available regional data and estimated the yearling birth rate parameter to be 1.09 fawns/yearling female based on the 5-year average of yearling fetal rates recorded by Wentworth et al. [28]. The fecundity of fawns was assumed to be zero, as our study and Wentworth et al. [28] observed no evidence of reproductive activity in fawns within the region. Including all parameters, the base model was as follows:

$$A=\left[\begin{array}{ccc} 0 & 0.519 & 0.597\\ 0.157 & 0 & 0\\ 0 & 0.952 & 0.835 \end{array}\right]$$

We lacked population estimation data, so initial abundances were simulated for each stage based on proportions calculated from the stable age distribution of a total population of 500 using observed vital rates. This initial population was broadly approximated based on the deer density estimate of 1.9–3.9 deer/km^2^ [13] in our 135 km^2^ study site. As a result, we used initial abundances equal to 192 fawns, 32 yearlings, and 276 adults. However, the accuracy of initial abundance values is not critical in stage-structured population models, as the estimated vital rate parameters determine the population growth rates λ used to develop conclusions [60].

We simulated five scenarios, parameterized with estimates from our study area and values reported in the literature (Table 1). We pooled estimated parameters across years (2018–2020) to reduce the effects of lower sample sizes among years. The base model used observed vital rates, and subsequent “what if” scenarios were variations of the base model. We incorporated stochastic survival rates for each life stage by repopulating matrices with values selected from a random uniform distribution within the 95% confidence intervals of the survival parameters for each annual time step [39]. We wanted all possible survival values to be equally likely on an annual basis to encapsulate the total extent of potential environmental effects (e.g., acorn crop fluctuation). We replicated this process 1000 times for each scenario to account for the full range of possible survival rate combinations and create more realistic scenarios [61,62]. We held fecundity parameters constant in each scenario, as described previously. We used sensitivity analysis to determine how individual vital rate parameters affect the rate of change in λ. This analysis uses the metrics of sensitivity and elasticity to determine the absolute contribution of each parameter on changes in λ and the proportional contribution of each parameter relative to the others, respectfully [63]. Due to use of stochastic model replication, we averaged growth rates λ and sensitivity metrics (i.e., sensitivity and elasticity) for each scenario to use in comparison. We did not use projected abundances as a metric of evaluation in this study because we lacked objective deer abundance estimates. All matrix analyses were completed using R software (version 4.1.3).

Scenario 1: Observed adult, yearling, and fawn survival—We established a base model using observed vital rates, which included hunter-related mortalities (*n* = 2) in the adult survival rate. Antlerless harvest was not permitted in the WMAs during the final year of the study. However, antlerless harvest opportunity was still available on surrounding private properties. The GDNR encouraged hunters not to avoid harvest of collared deer. Therefore, we considered the inclusion of these mortalities as representative of the level of harvest that currently occurs within our study area, which was 3% of our collared adults.

Scenario 2: No antlerless harvest and observed fawn survival—We omitted hunter-related mortalities to depict adult and yearling survival rates occurring in the absence of antlerless harvest on public and private lands, which also increased fecundity values. Robinson et al. [35] suggested that the reduction of antlerless harvest can compensate for most neonatal predation rates. However, our observed fawn survival rate was among the lowest recorded in the literature and a potential exception.

Scenario 3: 5% antlerless harvest and observed fawn survival—Chitwood et al. [38] and Peters et al. [39] approximated an annual antlerless harvest rate of 8–10% in their study areas. Antlerless harvest was likely lower in our study area during the last decade, as antlerless harvest opportunities were more restricted even prior to the new regulation eliminating antlerless harvest. Therefore, we chose an assumed antlerless harvest rate of 5% to evaluate the influence that reinitiating antlerless harvest on public land would have on population trajectory for comparison.

Scenario 4: Observed adult and yearling survival and moderate fawn survival—We incorporated fawn survival rates from comparable studies to represent the effects of increased fawn survival. Shuman et al. [17] reported a fawn survival rate of 0.270 (95% CI = 0.185–0.398) in a region of Louisiana with a similar predator composition as our study area, which was a representative average of rates recorded in the southeastern U.S. ranging from 0.141–0.430 [3,5,12,17,64]. Therefore, we used the same value as a description of moderate fawn survival.

Scenario 5: Observed adult and yearling survival and high fawn survival—The highest fawn survival rate recently reported in the southeastern U.S. with the use of VITs was 0.430 (95% CI = 0.290–0.570) in eastern Kentucky [64], a similar habitat to our study area. Therefore, we used that value to represent high fawn survival in this scenario.

## 3. Results

During 2018–2020, we collared 59 female white-tailed deer ≥ 1.5 years old (2018 = 12, 2019 = 23, 2020 = 24), including 14 yearlings (2018 = 2, 2019 = 5, 2020 = 7) and 45 adults (2018 = 10, 2019 = 18, 2020 = 17). We observed an annual adult survival rate of 0.835 (95% CI = 0.748–0.931; *n* = 45), with nine mortalities due to natural causes (2019 = 3, 2020 = 6) and two hunting-related deaths (2018 = 1, 2019 = 1). The annual adult non-hunting survival rate was 0.857 (95% CI = 0.772–0.950; *n* = 43). We did not observe any mortalities of our collared yearlings.

**Figure 2:**
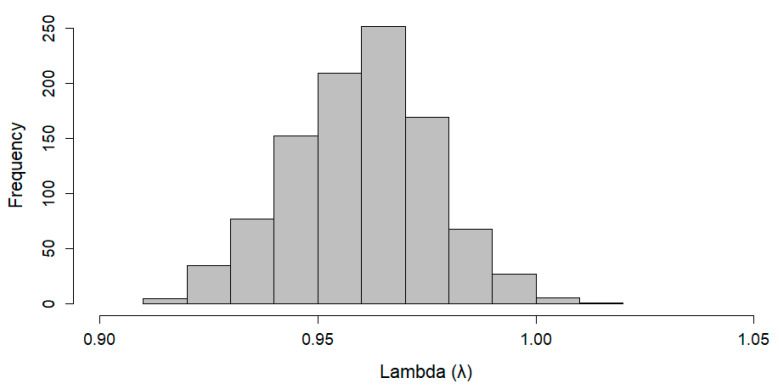
Frequency distribution of Scenario 1 growth rates (λ) for 1000 iterations of a female-only, stage-structured population model. The model was parameterized by observed white-tailed deer (*Odocoileus virginianus*) vital rates recorded in northern Georgia, USA, in 2018–2020, with an average annual population growth rate of λ = 0.960 (interquartile range (IQR) = 0.949–0.971).

**Figure 3:**
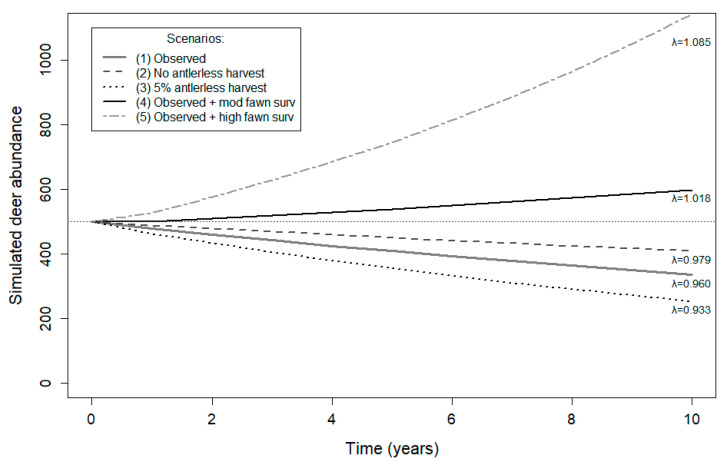
Population projections, including growth rates (λ), for different antlerless harvest and fawn survival scenarios over 10 years for female white-tailed deer (*Odocoileus virginianus*) in northern Georgia, USA, 2018–2020. Included scenarios: (1) observed fawn, yearling, adult survival; (2) no antlerless harvest and observed fawn survival; (3) 5% antlerless harvest and observed fawn survival; (4) observed yearling and adult survival and moderate fawn survival; (5) observed yearling and adult survival and high fawn survival.

Scenario 1 (observed vital rates) had a mean population growth rate of λ = 0.960 (interquartile range (IQR) = 0.949–0.971; Figure 2), indicating a 4.0% annual population decline (Figure 3). Scenario 1 predicted positive population growth in 1% of replicates. Scenario 2, which incorporated 0% antlerless harvest, had a mean population growth rate of λ = 0.979 (IQR = 0.969–0.990), projecting a 2.1% annual population decline and with only 10% of trajectories predicting positive growth. Scenario 3, which included 5% antlerless harvest, had a mean population growth rate of λ = 0.933 (IQR = 0.923–0.943), suggesting 6.7% annual population decline and with no projections predicting positive growth. Scenario 4 had a mean population growth rate of λ = 1.018 (IQR = 1.008–1.028), projecting 1.8% annual population growth and with 89% of trajectories predicting positive population growth. Lastly, Scenario 5 predicted the highest mean population growth rate of λ = 1.085 (IQR = 1.074–1.095), indicating 8.5% annual population growth and with 100% of trajectories projecting positive population growth. Annual growth rates across scenarios varied from a 6.7% decline to 8.5% positive growth. Adult female survival was the most sensitive and elastic parameter affecting change in λ in all scenarios, followed by the fawn survival parameter (Table 2). Under current conditions, using observed vital rate parameters, every 1% increase in adult survival resulted in an average λ increase of 0.008. A 1% increase in fawn survival resulted in an average λ increase of 0.006.

## 4. Discussion

Based on harvest data, white-tailed deer populations have declined drastically across the CNF in the Southern Appalachian region of northern Georgia since the early 2000s [13] despite more restrictive antlerless harvest regulations in the last decade. Our estimated vital rates and population trajectories were consistent with harvest data trends indicating decline. The results implied that continuing the prohibition of antlerless harvest in the WMAs is necessary to help curb population declines. However, current antlerless harvest restrictions would not wholly compensate for the low fawn survival rate in our study area (0.157; [4]), which is among the lowest rates recorded for white-tailed deer in North America [15]. Additionally, further reduction in antlerless harvest by eliminating opportunities on private lands does not seem likely to reverse population decline in our study region and could be subject to public contention. However, scenarios that incorporated moderate-to-high fawn survival rates in addition to current antlerless harvest restrictions on public lands projected positive population growth, which suggests the potential for population recovery.

Antlerless harvest can be an additive source of mortality in declining populations [65]; thus, the benefit of enforcing increasingly restrictive female harvest regulations (e.g., reduction of antlerless harvest days, decreased modern firearm opportunities). Robinson et al. [35] concluded that limiting antlerless harvest should be enough to combat the effects of coyote predation on fawns in eastern white-tailed deer populations, except in extreme cases of low recruitment. Our results provided one of those exceptions. Chitwood et al. [3] reported similar circumstances of low fawn survival (0.141) due to coyote predation on a site in North Carolina, concluding that antlerless harvest restrictions alone would not be enough to recover the population. In a special case, Bled et al. [66] documented a declining white-tailed deer population in South Florida with previous evidence of low recruitment that was also experiencing high adult mortality due to predation by a recovering Florida panther (*Puma concolor coryi*) population and periodic water inundation. They concluded that providing a sustainable deer harvest in the face of a large predator restoration is highly difficult, and deer hunting needed to be tightly regulated. The divergence of certain populations from the generalized conclusions of Robinson et al. [35] indicates the importance of obtaining site-specific estimates of vital rates when assessing declining deer populations.

The conclusions determined from matrix population models are highly dependent on the validity of vital rate estimations. Although, demographic estimates are inherently imprecise and may lack biological detail [50]. For instance, our models did not include the stochastic fecundity parameters that would be expected with real-world variation, especially for the yearling age class in our study region. Our study may be somewhat limited in this regard due to the absence of current yearling reproductive data. However, we feel confident in our substitutive use of the yearling fetal rate recorded by Wentworth et al. [28] in the same study region, as the parameter was averaged over five years of variable environmental conditions. Currently observed birth rates for the fawn and adult age classes were also similar to the fetal rate estimates of Wentworth et al. [28], offering further validation. We additionally believe our use of stochastic survival parameters incorporated over 1000 iterations for each model represented a wide range of possible outcomes, which were expressed in our results. Considering the difficulties in measuring exact vital rate parameters, matrix population models can still be used as a convenient summarizing tool for population growth rates and sensitivities [60] when parameters are representative of the species’ life history [50] like those used in our analysis.

In large mammal populations, adult female survival is commonly seen as the parameter most sensitive and elastic to effecting change in λ [32,36,67], but it typically varies little from year to year and among populations when excluding harvest [32,36]. We observed adult survival rates falling well within the range of recorded white-tailed deer survival rates across the eastern U.S. [58] and likely nearing the peak of what can be controlled by harvest regulations. Therefore, our observed adult survival rate will most likely remain stable; however, relying solely on antlerless harvest restrictions will not result in positive population growth. In contrast to the stability of adult female survival, fawn survival can be variable across years and regions, depending on environmental conditions. Due to this volatility, juvenile recruitment likely plays the predominant role in large herbivore population dynamics, even though offspring survival is generally found to be the second most sensitive vital rate [32,36,68]. We observed a low annual fawn survival rate across our study period (0.157), which is lower than reported from other regions [4]. Therefore, despite our results finding λ as most sensitive to change in adult survival, fawn survival is more likely to respond to management actions in our study system. If all vital rates were held constant, the annual fawn survival rate would theoretically need to be ≥0.256 for positive population growth to occur under observed conditions. Real-world practices seldom change vital rates following the exact proportions given by the sensitivity analysis, making it difficult to apply to management [69]. However, sensitivity analysis is still useful for identifying demographic parameters key to deer population viability [69] and directing management focus.

We conclude that low fawn survival and the related recruitment rates are the likely cause of population decline in the Southern Appalachian Mountain region of northern Georgia. Current fawn survival rates are likely dissimilar to historic rates, as active forest management has been dramatically reduced, thereby diminishing fawning cover [24]. In addition, coyotes were not in the region prior to the 1980s [18], and bears were rare in the Southern Appalachians until the 1970s [70]. Subsequently, annual bear population growth rates (λ) were estimated to be 1.07 and 1.08 for males and females, respectively, from 1979–2012 in our study region [13]. Bobcat populations have also increased [19], but their contribution to fawn mortality was found to be low [4]. The current fawn recruitment rate was estimated to be 0.22 fawns/female during 2018–2020 when incorporating fawn survival and birth rates [4], down from the 0.82–0.83 fawns/female estimate of 1983 [45]. The increase in predator numbers along with recent lack of forest disturbance have likely worked in synergy to increase fawn mortality. Mounting evidence has shown landscape configuration can have an impact on fawn predation rates. Fawns in contiguously forested habitats with minimal edge are at an increased risk of predation versus fawns in more diverse habitats [15,20,21,71]. The CNF is an expansive landscape of continuous forest lacking diversity, with a suite of predators that exemplifies an area expected to have high fawn predation rates.

The reported variability of fawn mortality rates across habitat types suggests that the promotion of heterogeneous landscapes could improve fawn survival [15]. Timber management combined with prescribed fire in closed-canopy hardwood forests can provide a patchwork of different seral stages and can facilitate understory growth, which is used for fawning cover and deer forage [72,73,74,75]. These habitat improvements would benefit fawn predator evasion [21] and maternal care [26]. However, the potential of habitat improvement through forest management to directly impact early fawn survival awaits substantiating research to inform population models, as the technique and scale of action likely influence effectiveness [76,77].

In the southeastern U.S., predator control has resulted in inconsistent responses in fawn survival rates of white-tailed deer. Howze et al. [8] and VanGilder et al. [9] found coyote and bobcat removals could be effective in increasing fawn recruitment in the short term. Kilgo et al. [10] and Gulsby et al. [11] found the impact of coyote removal on fawn survival to be variable across years and study sites due to the high immigration rates of coyotes [78], placing long-term effectiveness of coyote control in question. However, McCoy et al. [79] documented an example of higher fawn survival on an intensively managed study site with a long-term predator control program, although fawn survival may have been overestimated due to opportunistic fawn sampling. Regardless, removal efforts across a large landscape like the CNF raise questions concerning logistics and cost-effectiveness. In the western U.S., increased black bear harvest can increase elk (*Cervus canadensis*) calf survival. Tatman et al. [80] found that increasing spring black bear harvest to moderate and high levels just before calf-rearing season increased elk calf survival the following summer. White et al. [81] concluded that increasing both the fall and spring harvest of black bears and mountain lions (*Puma concolor*) had the potential to facilitate elk calf recruitment the following summer, but habitat variation needed to be considered. Spring black bear hunting does not occur in the southeastern U.S., so the effects of bear removal immediately prior to and during fawning are unknown. Additional bear harvest opportunities were initiated by the GDNR beginning in the fall 2020 season, authorizing the use of dogs for hunting [82]. However, further research to examine the impact that alternative black bear management, including increased harvest, would have on fawn survival of white-tailed deer in the eastern U.S. is needed.

## 5. Conclusions

Our study verified the occurrence of white-tailed deer population decline in our northern Georgia study site based on observed vital rates. The results further suggested antlerless harvest restrictions alone will not recover deer populations in northern Georgia, and efforts to increase fawn survival are necessary. Additional antlerless harvest restrictions on private lands are not advised, as such measures will not be enough to promote positive population growth and may conflict with preferences of the public. However, the antlerless harvest regulations implemented by the GDNR and the USFS on public lands are necessary and should remain in effect in concurrence with other management efforts. Supplementary measures such as habitat improvement and predator control should be explored. Forest management strategies to diversify forest-stage structure, increase edge, and enhance understory vegetation have the potential to reduce fawn predation risk and produce long-term results. Effective and financially feasible coyote management may be difficult across a large landscape, but strategies to curb bear population growth in the region through harvest, such as hunting with dogs, should be further examined in an effort to reduce fawn predation and stabilize deer populations.

The Southern Appalachian region is a unique environment that may not follow the general assumptions made of other North American white-tailed deer populations. We suggest analyzing population dynamics at a local scale, as deer vital rates, predator communities, and habitat conditions vary spatially. Responses of deer vital rates to specific management actions will therefore vary as well, with the scale of management activity potentially influencing effectiveness; thus, continued investigation of these relationships is recommended. Using field-based estimates of white-tailed deer vital rates to project future population trends has thus far been limited to a few studies. However, this approach can be used to approximate antlerless harvest prescriptions relative to various levels of fawn survival by managers throughout the Southern Appalachians and other regions to achieve desired population levels.

## Figures and Tables

**Table 1:** Annual survival (*S*) and fecundity (*F*) parameter values of white-tailed deer (*Odocoileus virginianus*) fawns (*f)*, yearling (*y*), and adults (*a*) used for multiple scenarios projecting female population growth over 10 years in northern Georgia, USA, 2018–2020. Survival parameters included 95% confidence intervals.

Scenario	Stage	Beta	Model Parameter
	Fawn (*F_f_*)		0
(1) Observed adult, yearling, and fawn survival	Yearling (*F_y_*) ^a^		0.519
	Adult (*F_a_*)		0.597
	Fawn (*S_f_*) ^b^	0.157	0.091–0.273
	Yearling (*S_y_*) ^c^	0.952	0.866–1.000
	Adult (*s_a_*)	0.835	0.748–0.931
(2) No antlerless harvest and observed fawn survival	Yearling (*F_y_*)		0.519
	Adult (*F_a_*)		0.613
	Fawn (*S_f_*)	0.157	0.091–0.273
	Yearling (*S_y_*)	0.952	0.866–1.000
	Adult (*S_a_*)	0.857	0.772–0.950
(3) 5% antlerless harvest and observed fawn survival	Yearling (*F_y_*)		0.519 × 0.95
	Adult (*F_a_*)		0.597 × 0.95
	Fawn (*S_f_*)	0.157	0.091–0.273
	Yearling (*S_y_*)	0.904	(0.866–1.000) × 0.95
	Adult (*S_a_*)	0.814	(0.772–0.950) × 0.95
(4) Observed adult and yearling survival and moderate fawn survival	Yearling (*F_y_*)		0.519
	Adult (*F_a_*)		0.597
	Fawn (*S_f_*) ^d^	0.270	0.185–0.398
	Yearling (*S_y_*)	0.952	0.866–1.000
	Adult (*S_a_*)	0.835	0.748–0.931
(5) Observed adult and yearling survival and high fawn survival	Yearling (*F_y_*)		0.519
	Adult (*F_a_*)		0.597
	Fawn (*S_f_*) ^e^	0.430	0.290–0.570
	Yearling (*S_y_*)	0.952	0.866–1.000
	Adult (*S_a_*)	0.835	0.748–0.931

^a^ Derived from data reported in Wentworth (1990) [28]. ^b^ Reported in Edge et al. (2023) [4]. ^c^ Reported in Peters et al. (2020) [39]. ^d^ Reported in Shuman et al. (2017) [17]. ^e^ Reported in McDermott (2017) [64].

**Table 2:** Sensitivities and elasticities of white-tailed deer (*Odocoileus virginianus*) vital rate parameters (i.e., fecundity and survival) at different age classes (i.e., fawn, yearling, and adult) used to project female population growth over 10 years for multiple scenarios in northern Georgia, USA, 2018–2020.

Scenario	Parameter	Sensitivity	Elasticity
(1) Observed adult, yearling, and fawn survival	Yearling fecundity	0.022	0.012
	Adult fecundity	0.157	0.097
	Fawn survival	0.586	0.109
	Yearling survival	0.100	0.097
	Adult survival	0.783	0.685
(2) No antlerless harvest and observed fawn survival	Yearling fecundity	0.020	0.011
	Adult fecundity	0.152	0.095
	Fawn survival	0.579	0.106
	Yearling survival	0.100	0.095
	Adult survival	0.788	0.693
(3) 5% antlerless harvest and observed fawn survival	Yearling fecundity	0.022	0.012
	Adult fecundity	0.158	0.096
	Fawn survival	0.565	0.108
	Yearling survival	0.101	0.096
	Adult survival	0.785	0.689
(4) Observed adult and yearling survival and moderate fawn survival	Yearling fecundity	0.042	0.021
	Adult fecundity	0.211	0.124
	Fawn survival	0.520	0.145
	Yearling survival	0.135	0.124
	Adult survival	0.710	0.586
(5) Observed adult and yearling survival and high fawn survival	Yearling fecundity	0.072	0.034
	Adult fecundity	0.264	0.145
	Fawn survival	0.461	0.179
	Yearling survival	0.169	0.145
	Adult survival	0.641	0.496

## Data Availability

All relevant data can be found within the article and its Supplementary Materials.

## Supplementary Materials

The following supporting information can be downloaded at: https://www.mdpi.com/article/10.3390/ani13233675/s1, Table S1: White-tailed Deer Yearling and Adult Survival Data: Northern Georgia, USA (2018–2020).
