# Supplementary material for: Population Dynamics of a Declining White-Tailed Deer Population in the Southern Appalachian Region of the United States
Source: Animals (Basel). 2023 Nov 28;13(23):3675. doi: 10.3390/ani13233675 (PMC10705329; doi:10.3390/ani13233675)
Supplement: Supplementary file 1 [file animals-13-03675-s001.zip › animals-2710039-supplementary.pdf]

White-tailed Deer Yearling and Adult Survival Data

Northern Georgia, USA (2018–2020)

University of Georgia – Warnell School of Forestry and Natural Resources

Deer ID	Study entry (Julian date)	Study exit (Julian date)	Mortality Event	Mortality cause	Adult (>2yrs)	Yearling (1-2yrs)	Study Year	Collar notes
004	14	273	Yes	Hunter harvest	Yes	No	2018	
005	23	365	No	None	Yes	No	2018	
005	1	365	No	None	Yes	No	2019	
005	1	162	No	None	Yes	No	2020	Removed
003	39	365	No	None	Yes	No	2018	
003	1	365	No	None	Yes	No	2019	
003	1	365	No	None	Yes	No	2020	
010	46	365	No	None	No	Yes	2018	
010	1	365	No	None	Yes	No	2019	
010	1	365	No	None	Yes	No	2020	
007	55	365	No	None	Yes	No	2018	
007	1	365	No	None	Yes	No	2019	
007	1	365	No	None	Yes	No	2020	
022	58	365	No	None	Yes	No	2018	
022	1	78	No	None	Yes	No	2019	Removed
012	59	365	No	None	Yes	No	2018	
012	1	360	Yes	Hunter harvest	Yes	No	2019	
052	63	365	No	None	Yes	No	2018	
052	1	365	No	None	Yes	No	2019	
052	1	39	Yes	Natural causes	Yes	No	2020	
024	72	365	No	None	Yes	No	2018	
024	1	365	No	None	Yes	No	2019	
024	1	365	No	None	Yes	No	2020	
009	72	365	No	None	No	Yes	2018	
009	1	365	No	None	Yes	No	2019	
009	1	365	No	None	Yes	No	2020	
019	75	365	No	None	Yes	No	2018	
019	1	149	No	None	Yes	No	2019	Removed
018	81	365	No	None	Yes	No	2018	
018	1	365	No	None	Yes	No	2019	
018	1	220	No	None	Yes	No	2020	Removed
071	13	13	No	None	Yes	No	2019	Malfunction
076	13	169	Yes	Natural causes	Yes	No	2019	
072	13	365	No	None	No	Yes	2019	
072	1	365	No	None	Yes	No	2020	
095	16	365	No	None	No	Yes	2019	
095	1	64	Yes	Natural causes	Yes	No	2020	
062	24	365	No	None	Yes	No	2019	
062	1	365	No	None	Yes	No	2020	
093	25	365	No	None	Yes	No	2019	

093	1	365	No	None	Yes	No	2020	
081	26	365	No	None	No	Yes	2019	
081	1	365	No	None	Yes	No	2020	
087	31	257	Yes	Natural causes	Yes	No	2019	
086	32	365	No	None	Yes	No	2019	
086	1	365	No	None	Yes	No	2020	
088	38	365	No	None	Yes	No	2019	
088	1	85	Yes	Natural causes	Yes	No	2020	
085	40	365	No	None	Yes	No	2019	
085	1	365	No	None	Yes	No	2020	
096	41	365	No	None	Yes	No	2019	
096	1	365	No	None	Yes	No	2020	
100	42	365	No	None	Yes	No	2019	
100	1	365	No	None	Yes	No	2020	
105	42	109	Yes	Natural causes	Yes	No	2019	
103	44	365	No	None	Yes	No	2019	
103	1	365	No	None	Yes	No	2020	
099	56	365	No	None	Yes	No	2019	
099	1	365	No	None	Yes	No	2020	
013	65	365	No	None	No	Yes	2019	
013	1	365	No	None	Yes	No	2020	
101	66	365	No	None	No	Yes	2019	
101	1	365	No	None	Yes	No	2020	
104	66	365	No	None	Yes	No	2019	
104	1	365	No	None	Yes	No	2020	
063	82	365	No	None	Yes	No	2019	
063	1	365	No	None	Yes	No	2020	
102	82	365	No	None	Yes	No	2019	
102	1	2	Yes	Natural causes	Yes	No	2020	
097	82	365	No	None	Yes	No	2019	
097	1	365	No	None	Yes	No	2020	
014	86	365	No	None	Yes	No	2019	
014	1	219	Yes	Natural causes	Yes	No	2020	
113	5	42	Yes	Natural causes	Yes	No	2020	
187	5	365	No	None	Yes	No	2020	
111	12	51	No	None	Yes	No	2020	Removed
185	17	365	No	None	No	Yes	2020	
189	18	365	No	None	Yes	No	2020	
114	21	365	No	None	No	Yes	2020	
199	21	365	No	None	Yes	No	2020	
206	21	365	No	None	Yes	No	2020	
188	24	323	No	None	No	Yes	2020	Malfunction
193	26	365	No	None	No	Yes	2020	
196	26	365	No	None	Yes	No	2020	
191	27	365	No	None	Yes	No	2020	
011	30	365	No	None	Yes	No	2020	
108	48	365	No	None	No	Yes	2020	
169	54	365	No	None	Yes	No	2020	

181	54	54	No	None	Yes	No	2020	Malfunction
201	54	365	No	None	Yes	No	2020	
195	56	365	No	None	Yes	No	2020	
200	56	365	No	None	Yes	No	2020	
160	63	92	No	None	Yes	No	2020	Removed
117	70	365	No	None	Yes	No	2020	
116	75	365	No	None	Yes	No	2020	
507	77	108	No	None	No	Yes	2020	Removed
510	77	109	No	None	No	Yes	2020	Removed
